# Song Preference in Female and Juvenile Songbirds: Proximate and Ultimate Questions

**DOI:** 10.3389/fphys.2022.876205

**Published:** 2022-04-14

**Authors:** Tomoko G. Fujii, Austin Coulter, Koedi S. Lawley, Jonathan F. Prather, Kazuo Okanoya

**Affiliations:** ^1^ Department of Life Sciences, Graduate School of Arts and Sciences, The University of Tokyo, Tokyo, Japan; ^2^ Department of Zoology and Physiology, Program in Neuroscience, University of Wyoming, Laramie, WY, United States; ^3^ Department of Biomedical Sciences, Texas A&M University, College Station, TX, United States

**Keywords:** birdsong, song preference, mate choice, song tutor choice, vocal learning, auditory learning, neural circuit

## Abstract

Birdsong has long been a subject of extensive research in the fields of ethology as well as neuroscience. Neural and behavioral mechanisms underlying song acquisition and production in male songbirds are particularly well studied, mainly because birdsong shares some important features with human speech such as critical dependence on vocal learning. However, birdsong, like human speech, primarily functions as communication signals. The mechanisms of song perception and recognition should also be investigated to attain a deeper understanding of the nature of complex vocal signals. Although relatively less attention has been paid to song receivers compared to signalers, recent studies on female songbirds have begun to reveal the neural basis of song preference. Moreover, there are other studies of song preference in juvenile birds which suggest possible functions of preference in social context including the sensory phase of song learning. Understanding the behavioral and neural mechanisms underlying the formation, maintenance, expression, and alteration of such song preference in birds will potentially give insight into the mechanisms of speech communication in humans. To pursue this line of research, however, it is necessary to understand current methodological challenges in defining and measuring song preference. In addition, consideration of ultimate questions can also be important for laboratory researchers in designing experiments and interpreting results. Here we summarize the current understanding of song preference in female and juvenile songbirds in the context of Tinbergen’s four questions, incorporating results ranging from ethological field research to the latest neuroscience findings. We also discuss problems and remaining questions in this field and suggest some possible solutions and future directions.

## 1 Introduction

Birdsong has been extensively studied in multiple disciplines that address animal behavior. A large body of field and laboratory work in songbirds (order Passeriformes, suborder Passeri) has revealed that songs are typically used for courtship and territorial defense (Catchpole and Slater, 2008). Because of historical and geographical biases, function of birdsong is particularly well studied in species where only males sing to repel male rivals or to attract female mates, although song is not a male-specific trait in a substantial number of species (Odom et al., 2014; Riebel, 2016). Since the finding of learned aspects of vocal behavior such as song dialects and cultural transmission (Marler and Tamura, 1964), great effort has been devoted to elucidating the mechanisms of song learning in males (Brainard and Doupe, 2002; Bolhuis and Gahr, 2006; Mooney, 2009; Ikeda et al., 2020). Now, growing evidence shows that the process of song development in songbirds is quite similar to that of speech acquisition in humans, and these similarities are evident at behavioral, neural, and genetic levels (Doupe and Kuhl, 1999; Bolhuis et al., 2010; Prather et al., 2017). Partly due to these shared features with human speech, neuroethology of birdsong has primarily focused on the signalers. As a result, neural mechanisms of song learning and production in males have been more intensively studied than those of song perception and recognition in either sex (Hernandez et al., 2008; Riebel, 2009).

Birdsong, like human speech, is used to convey information. Although relatively less attention has been paid to song receivers compared to signalers, investigation into the mechanisms of song perception and recognition should also be an important focus as we seek to attain a deeper understanding of vocal behavior. It has been demonstrated in several species that adult and juvenile birds of both sexes can discriminate songs of different categories or acoustic and temporal features (Searcy and Yasukawa, 1996; Riebel, 2009; Rodríguez-Saltos, 2017). Their sensitivity to differences between song stimuli has been measured by behavioral, physiological, and molecular methods, and their acuity suggests the ability to recognize species, local populations, and individuals based on song features (Miller, 1979; Dooling and Searcy, 1980; Clayton, 1988; Gentner and Hulse, 2000; Maney et al., 2003; Woolley and Doupe, 2008). Especially in case of females, such song discrimination abilities have been explored in the theme of mate choice. In a series of studies both in the field and laboratory, selectivity of behavioral responses to one song stimulus over others is often called “song preference.” For instance, if female birds show more frequent responses to conspecific songs than to heterospecific songs, such selective responses are described as a preference for songs of their own species, and those preferences are interpreted as evidence that the females are more likely to mate with conspecific males based on their songs (Wagner, 1998).

Female song preference has traditionally been studied in the field of behavioral ecology, but recent studies on female songbirds have also begun to reveal the neural basis of song preference, as detailed in subsequent sections. Moreover, there are other new studies of song preference in juvenile birds which suggest possible functions of preference in learning associated with song production. Neuroethological studies on these topics have just begun to emerge, and there is still much to explore, including how song preference is formed, maintained, expressed, and altered. To pursue this line of research, however, it is important to grasp current methodological challenges in defining and measuring song preference. In addition, consideration of ultimate questions (i.e. functions in reproductive success and evolution of song preferences) should also be helpful for laboratory researchers in designing experiments and interpreting results. To clarify the problems and discuss future directions, here we summarize the current understanding of song preference in female and juvenile songbirds in the context of Tinbergen’s four questions, incorporating results ranging from ethological field research to the latest neurobiological findings.

## 2 What Is Song Preference

### 2.1 Terminology and Definition

“Preference” is a term that is used very commonly and in many contexts. Its flexibility of use is evident in several different research contexts even within the field of animal behavior. The definition of song preference based on the act of measurement sometimes differs from one study to another. To better understand how the term “song preference” is defined either conceptually or operationally, here we briefly summarize the usage and definition of the term.

“Song preference” has been used in studies of female mate choice where it is postulated that female birds select a suitable mate among multiple males they encounter. In addition to a range of features that provide insight into a suitor’s fitness, such as plumage, song is an especially important element in how females evaluate the quality of male birds. Thus, investigating how females respond to within- or between-species song variation is fundamental to understanding of songbird mating preference and mate choice. Here, mating preference denotes a disposition or propensity that a female possesses, while mate choice is the manifestation of preference which is also affected by other conditions including external factors such as social environment and habitat structure (Jennions and Petrie, 1997; Widemo and Sæther, 1999; Cotton et al., 2006; Kirkpatrick et al., 2006). Song preference can be regarded as one aspect of mating preference, because morphological and behavioral traits other than song are also available for mate evaluation (Collins and Ten Cate, 1996; Grant and Grant, 1997; Brazas and Shimizu, 2002; Gomes et al., 2017). The term “song preference” is also used in studies of song tutor choice in juvenile males who are about to memorize and imitate songs from conspecific adults. Song preference in juveniles potentially enable them to selectively attend to an appropriate song model among multiple options, but the preference for a tutor may also be influenced by other behaviors and traits of the adults or environmental factors (Clayton, 1987; Soma et al., 2009). More background will be reviewed in another section, but the basic idea regarding juvenile song preference is similar to that of females in that both focus on how birds respond to different songs and use those experiences to shape their subsequent behavior. In summary, song preference is a tendency or likelihood for an individual to respond to one song among multiple song samples that the bird may hear, and preference for a song has considerable relevance to the preference for the associated singer. These preferences predict future behavior, but they do not prevent departure from those preferences. Thus, one must keep in mind that preferences bias behavior, but there can be a difference between perception of a preference and expression of the associated choice.

Whatever the underlying concept of the term, such as females choosing a mate or males selecting a tutor to imitate, researchers need a means to operationally define song preference in scientific studies. To operationally define song preference, we need observable motor responses to song stimuli; and we need ways to measure and compare those responses to different stimuli. In the field of animal behavior, an animal’s response to a stimulus (e.g., approaching food or avoiding predators) is generally interpreted as an expression of the animal’s motivation (Berridge, 2004). Accordingly, preference denotes a difference between the strength of motivation to obtain or avoid one stimulus over others (Fraser and Matthews, 1997; Kirkden and Pajor, 2006). Thus, an experimenter can quantify the animal’s preference by playing alternative sound stimuli with a suitable presentation (Wagner, 1998) and comparing the frequency, duration, or intensity of a given behavior that is produced in response. Songbird researchers have found several types of behaviors that are available to quantify preferences in laboratory playback studies (Searcy, 1992).

Taken together, song preference in birdsong ethology and neuroscience is operationally regarded as the behavioral response selectivity for one song over others, and this preference is assumed to underlie the process of mate choice or tutor choice at a conceptual level. While accepting the operational definition in practice, it is also important to consider what the bird may actually be perceiving and indicating in their behavioral indicators of preference that are measured in experiments. Because it is important to be aware of strengths and weaknesses of each type of behavioral measurements, we consider several examples in the following subsection.

### 2.2 How to Measure Song Preference

In attempts to quantify and understand cognitive behaviors such as mate preference, it can be challenging to assess an organism’s performance. It can be challenging to develop tests that reveal a female songbird’s preference for the features of individual songs and their intention to choose the associated singer as their mate. In such studies, researchers must be careful to control for other features of a male bird’s behavior or other characteristics, such as the quality of a male’s plumage or a male’s expression of other courtship behaviors. This exclusion of facets of communication other than song can be accomplished by recording the songs produced by male birds and playing them through a speaker when no male is physically present. Song is an especially impactful stimulus in female evaluation of the quality of the male suitor, and song is sufficient to drive female courtship and copulatory behaviors even when no male is present (Woolley and Doupe, 2008; Caro et al., 2010; Dunning et al., 2014, Dunning et al., 2020; Heinig et al., 2014; Perkes et al., 2019, Perkes et al., 2021; Fujii and Okanoya, 2022). Thus, song is a unimodal stimulus that facilitates investigations of how the qualities of that stimulus affect female evaluation of the quality of those signals. The remaining challenges center on how to assess female responses and interpret the relation between those behaviors and a female’s preferences. Researchers have sought to develop ways to detect evidence of a bird’s cognitive behaviors such as song perception and evaluation.

Many paradigms have been employed to assess female song preference, and these can be broadly categorized as operant versus naturalistic response to song stimuli. In both paradigms, a variety of songs are typically presented to a female bird while observing her immediate responses to those stimuli. To avoid pseudoreplication, many songs should be recorded from each male so that each is represented by many exemplars of his song performances. The difference between operant and naturalistic approaches lies in the nature of the responses that the female displays. In operant conditioning, rewards or punishments are used to train a bird to express a specific behavior in response to songs that they perceive as having a specific characteristic. For example, a bird may be taught to express one behavior when she hears a song that she recognizes as familiar or finds very attractive, and she may be taught to express another behavior in response to a song that is novel or that she finds less attractive (Burt et al., 2000). These sorts of operant behavioral responses can include things such as hopping onto one perch or another, pulling a string, or pecking a target. Often, some form of reward or punishment is used to motivate the behavior, such as food or brief periods of darkness, respectively. A distinct advantage of studying female songbirds is that song appears to be inherently rewarding, such that the ability to induce song playback is sufficient to motivate the females to engage in behavior (e.g., Riebel and Slater, 1998). This facilitates experimentation by making it relatively easy to induce the birds to engage in the associated behavior, and it removes a possible confound to interpreting the nature of the preference that the female is revealing in her responses.

The advantages of operant paradigms are significant, but an operant approach commonly requires subjects to engage in some behavior that is different than they would do in response to songs heard in the natural environment. This has led some researchers to measure female preference by observing naturalistic behaviors that females perform in response to song stimuli. These behaviors include things such as adopting the unusual posture that defines a courtship solicitation display (CSD), calling in different amounts in response to different stimuli, approaching one speaker or another, or orienting their body toward the source of sound in a behavior called phonotaxis (e.g., Caro et al., 2010) As in the case of operant conditioning, there are both advantages and disadvantages to these more naturalistic approaches. For example, the fact that females express behaviors naturally means that researchers can avoid concerns inherent to the training and reward processes used in operant conditioning. However, naturalistic studies are not immune to difficulties in interpreting the meaning of a female’s behavior. For example, a female may approach a speaker for a number of different reasons, such as seeking to hear the song more clearly, seeking to associate with something they recognize as simply familiar, or seeking to investigate the source of a song that they find attractive as a courtship signal. In each case, the observed behavior (approach) is the same, but the underlying motivation could be quite different. The female could be seeking to gain more information in their assessment, the safety of associating with a particular individual, or a reproductive opportunity in response particularly attractive song. These potential confounds emerge in any condition in which researchers are seeking to interpret the actions of an experimental subject that can’t clearly communicate its intent. One idea is that some combination of operant and naturalistic behaviors could be the best way to measure female song preference, but it remains unknown what method or combination of methods may be the most revealing and least confounding way to assess that perception.

Some researchers have used the expression of CSD’s as the ultimate indicator that a female finds a signal attractive (e.g., Anderson, 2009; Dunning et al., 2014). Reports have revealed congruence between the expression of CSDs and tests performed on the same birds using either operant approaches or measures of naturalistic behaviors (e.g., Anderson, 2009; Dunning et al., 2014). These results support the idea that each of these approaches is a valid behavioral measure of song preference; however, the robust expression of CSD’s often requires an altered state, such as subcutaneous implants to induce elevated levels of hormones such as estradiol (reviewed in Maney, 2010; see also Maney et al., 2008). This is especially important for researchers seeking to understand the neural mechanisms that underlie song perception and evaluation, as the presence or absence of estradiol can have a profound impact on auditory responses of neurons that are thought to play essential roles in those cognitive processes (Maney et al., 2003, Maney et al., 2008; Krentzel et al., 2018, Krentzel et al., 2020; Lee et al., 2018). A state-dependent difference in patterns of neural activity gives rise to the possibility that the mechanisms and patterns of activity that are at work in one state could be different than those at work in another state (i.e., artificially elevated levels of estradiol versus physiological levels). An additional complication is that the effects of estradiol are not constant over time, with implants commonly inducing robust expression of CSDs for only a brief window of time (discussed in Anderson, 2009). These complications are largely unavoidable in the experimental process, and they point to the value of multiple approaches to measure female song preference and mate choice. An important goal of future studies should be to reconcile data from these multiple approaches and physiological states as they seek to discern general principles that underlie the processes of song perception and decision-making in the natural condition.

## 3 Female Song Preference

### 3.1 Ultimate Questions

#### 3.1.1 Function

To understand the mechanisms and evolutionary development of song preferences, it is important to understand how they may provide benefit for songbirds. Song performances not only provide the listeners insight into the quality of the associated singer but serve many purposes, both within and outside of a courting pair. One of the primary functions of song preference in songbirds is to identify the singer. Song evaluation and preference allow the listener to determine whether the singer is a member of their own species (conspecific) or another species (heterospecific). When given the choice, females across species prefer the songs of conspecifics over those of heterospecifics (Clayton and Pröve, 1989; Hernandez and MacDougall-Shackleton, 2004; Diez et al., 2019). This preference for conspecifics helps to facilitate social interactions and courtship behaviors with members of their own group.

Male behavior also facilitates those social interactions, with males engaging in courtship behaviors to different degrees around different females (Heinig et al., 2014). For example, males can perform songs in association with overt bodily orientation and hopping back and forth as a signal to the female that the song performance is being directing toward her (Frith and Frith, 1988). These “directed songs” are acoustically more precise than other “undirected songs” performed either in isolation or in groups without any overt orientation toward a specific receiver (Riters and Stevenson, 2012). Female songbirds prefer those directed song performances more than undirected songs from the same individual (Woolley and Doupe, 2008; Riters and Stevenson, 2012; Dunning et al., 2014; Heinig et al., 2014; Schubloom and Woolley, 2016). Exposure to songs that the female finds attractive is thought to facilitate the physiological processes associated with successful mating, allowing her preference to directly impact reproductive success (Slater and Mann, 2004). This is especially clear in the case to birds that live in tropical climates, where courtship behaviors are thought to play an even greater role than photoperiod or other seasonal shifts in affecting reproductive status (e.g., Hoffmann et al., 2019). During successful courtship by a male, the female engages in a duet by singing along with him in remarkable temporal precision (discussed in Elie et al., 2019). Together, these studies from tropical climates, where reproductive status is most strongly influenced by behavioral cues, and temperate climates, where reproductive status is shaped by both environmental and behavioral cues, offer opportunities to understand the mechanisms that underlie the expression of song preferences in mate choice.

In addition to recognition of species identity, songs provide sufficiently high-resolution information that birds can identify individuals from the same local area and even the same family. Females of many species prefer songs that are performed by members of their local population over songs performed by males from other populations (Spitler-Nabors and Baker, 1983; O’Loghlen and Rothstein, 1995; Le Maguer et al., 2021). Such preferences are thought to confer benefit to the females by enabling them to mate assortatively, permitting social, geographic, and genetic stability within their population (Spitler-Nabors and Baker, 1983). Studies from many species have shown that females prefer their father’s song over unfamiliar songs from males of the same species (Miller, 1979; Clayton, 1988; Fujii et al., 2021; Le Maguer et al., 2021). The nature of that preference has remained unclear, as females will approach or otherwise choose to interact with the source of that song, but it is not clear what sort of intent might be associated with that. For example, the female could be seeking familiarity, or seeking the protection of their family group. A recent study showed that the father’s song can be a strong stimulus in eliciting CSDs in female Bengalese finches. (Fujii and Okanoya, 2022). If the father’s song is consistently the female’s most preferred song, that could have detrimental effects on fitness. Many additional aspects of a female’s developmental history also affect their song preference. The female hears her father’s song many times throughout development, and a relation between preference and the father’s song is consistent with the idea that the juvenile’s experience of their father’s song could possibly help them form a mental model of what a high-quality song sounds like (Miller, 1979; Clayton, 1988; Fujii et al., 2021).

Song preferences are also thought to confer advantage by allowing female to evaluate the quality of male suitors as potential mates (reviewed in Catchpole and Slater, 2008). Two key challenges in research involving those preferences have been identifying which features of song are most salient in communicating quality and determining whether quality of song performance may be associated with other more general aspects of cognitive ability. Although this latter idea has been alluring and persistent, many studies have found no consistent relationships between song ability and other forms of cognitive ability (Templeton et al., 2014; Anderson et al., 2017). Nonetheless, song may provide insight into the life history of the singer and thus provide insight into his potential quality as a mate. If a male experiences nutritional stress during juvenile development, that negative experience can have a similarly negative impact on many facets of development, including learned song performance (O’Loghlen and Rothstein, 1995; Nowicki et al., 2002; Lauay et al., 2004; Chen et al., 2017; Bircher and Naguib, 2020). Females are able to detect those subtle impacts, and they express greater preference for songs performed by normally reared birds than for their nutritionally stressed counterparts (reviewed in Nowicki et al., 1998, Nowicki et al., 2002). This is thought to provide benefit by helping females avoid nutritionally compromised and developmentally challenged mates. This idea is also reflected in the reduced female preference for songs performed by males that are experiencing infection or other ailments that engage the immune system (Garamszegi et al., 2004; Spencer et al., 2005; Dreiss et al., 2008; Munoz et al., 2010).

Just as female preference for song can be affected by the status of the singer, the female’s preference can also be affected by her own status. For example, one study raised broods of songbirds under a variety of conditions to create what they termed high-quality (good nutrition and parental care) and low-quality (poor nutrition and parental care) offspring (Burley and Foster, 2006). When queried about their mate preferences, high-quality females tended to prefer songs of high-quality males. When the females’ status was negatively impacted by trimming the most distal portion of their feathers, their preferences tended to change to now prefer the songs of low-quality males (Burley and Foster, 2006; Holveck and Riebel, 2010; Holveck et al., 2011). Thus, care must be taken when interpreting data regarding mate preferences, as they can vary not only across individuals but also within an individual tested at different in different conditions.

The impact of female mate preferences goes beyond the initial mating ritual, affecting many aspects of a pair’s mate bond. This is evident in the greater likelihood of success for pairings in which females select the partner whose song she prefers. When this is not the case, such as instances when mate pairs are selected arbitrarily by breeders, the number and health of the offspring are less than when the female’s choice is the prime determinant of the pairing (Schubloom and Woolley, 2016). The role of female preference is also evident in pair maintenance long after the initial pairing. For example, females that have mated with males that perform high-quality songs are less likely to seek extra-pair copulations, while females that have mated with low-quality males are more likely to seek extra-pair copulations (Garamszegi et al., 2004; Chiver et al.,2008; Byers and Kroodsma, 2009). Together, these data make clear the role of mate preferences of both female and male birds in what is ultimately a mutual mate choice, and they lead to questions about how those preferences have emerged through evolution and how they are expressed throughout ontogeny.

#### 3.1.2 Evolution

Questions about the evolution of female song preference is of great scientific significance for neurobiologists who study the mechanisms underlying song preference. Explanations for the evolution of mating preference are mostly based on direct or indirect benefit to individuals as a result of their mate choices, or sensory or perceptual biases that affect the receivers in general contexts (Jennions and Petrie, 1997; Kokko et al., 2003; Ryan, 2021). Both these are general and well-established theoretical frameworks, but in case of birdsong research, particularly the former one has been the target of intensive discussion and empirical examination. Reconstructing the evolutionary process of a certain preference is a challenging task, but it can be addressed by estimating the fitness costs and benefits of possessing the preference and/or by comparing the preference among multiple extant species with analysis of the phylogenetic relationship. Here, we take up some examples where the evolutionary background of the preference has been investigated through these approaches.

Just as there is variation between species in what song features they prefer, there is also variation even within species such that different females may have different profiles of song preference. Nonetheless, years of research have revealed some song features that are broadly impactful across members of a species, and even across different species, and are thus significant influences on female birds’ responses. Typically, females prefer songs of higher output, such as longer duration, higher frequency, or larger amplitude (Nowicki and Searcy, 2004; Catchpole and Slater, 2008). Because longer or louder singing requires more time and energy and should also be conspicuous to predators, song output is thought to be a reliable indicator of male signaler (Nowicki and Searcy, 2005; MacDougall-Shackleton and Spencer, 2012). There is empirical data showing correlation between song output and male reproductive success or other indices of male quality (Gil and Gahr, 2002; Catchpole and Slater, 2008), which supports the assumption of this signal honesty.

In addition, preference for song complexity has been commonly demonstrated in several species (Searcy and Yasukawa, 1996; Catchpole and Slater, 2008). Complexity in this context can refer to the number of song types in a male bird’s repertoire, or the size of song note repertoire. In contrast to song output, it is not apparent whether and how the song complexity can be a reliable indicator of male quality. However, an idea proposed as the “developmental stress hypothesis” (Nowicki et al., 1998, Nowicki et al., 2002) provides a clue to solve this puzzle. A key point of this hypothesis is that male birds learn their songs as juveniles, and they are particularly susceptible to nutritional or environmental stress during that time, though the degree of stress varies across species (c.f., Russell et al., 2004). It is thought that individuals who are resistant to such stress or suffer less stress can allocate metabolic resources to the development of neural structures for motor or cognitive abilities including singing (Crino and Breuner, 2015). In this way, song quality of an adult male is thought to be correlated with other traits that are also affected by stress during one’s developmental history. Substantial number of studies support the predictions of the hypothesis, such as the effect of stress on the behavior or neural structure in males, for example in song sparrows, starlings, and canaries (reviewed in MacDougall-Shackleton, 2015; MacDougall-Shackleton and Spencer, 2012). Nonetheless, other reports indicate little correlation between aspects of song performance and features of general cognition, so understanding what benefit may be gleaned from selecting a mate with a complex song remains a topic of ongoing research (Anderson et al., 2017; DuBois et al., 2018).

Another specific aspect of song complexity that varies across and within species is the transitional pattern of song notes (Okanoya, 2004a; Soma et al., 2006a). It was previously found that Bengalese finches’ songs contain complex transition patterns (Honda and Okanoya, 1999; Katahira et al., 2013), and that a certain population of females prefer more complex songs than simpler songs (Okanoya and Takashima, 1997; Morisaka et al., 2008; Kato et al., 2010). In addition, early developmental condition affects the sequential complexity of the song and the body size of males (Soma et al., 2006b), supporting the possibility that the preference for sequential complexity is also explained by the developmental stress hypothesis.

There are some caveats when considering possible explanations for potential benefits from selecting a mate based on specific aspects of learned song performance. First, the manipulation of developmental conditions in a laboratory may not exactly mimic the stresses that birds encounter in the natural environment. Therefore, balance between experimental control and external validity of methodology should be considered (MacDougall-Shackleton, 2015). Second, male song complexity may not only be influenced by female preferences but also advantages of signal modulation in managing social interactions with diverse individuals through song, including between-males song matching (Byers and Kroodsma, 2009). Also, some people indicate that the cost of having mating preferences is much less explored than the benefit, despite its importance (Jennions and Petrie, 1997). In this regard, investigation into the neural mechanisms that enable evaluation of song complexity may shed light on the cognitive cost, and eventually the evolutionary process of the song preference.

### 3.2 Proximate Questions

#### 3.2.1 Development

Similar to how male birds learn their songs from the sounds they hear during development, female song preference is also shaped by juvenile experience. For example, female songbirds commonly prefer songs performed by members of their local population more than songs performed by males from a different population (Spitler-Nabors and Baker, 1983; O’Loghlen and Rothstein, 1995; Le Maguer et al., 2021). This suggests that females may prefer songs that they heard during development, but it leaves open the question of whether that preference is learned or related to some genetic predisposition. A study of female swamp sparrows addressed this by collecting hatchling birds from the wild and rearing them in the laboratory under carefully controlled acoustic conditions. When the females reached adulthood, they expressed a preference for the songs that they had heard during development (Anderson et al., 2014). Importantly, the females’ preference for familiar songs from their local population was much greater than their responses to songs that were also recorded from their local population but were not played to them during juvenile development (Anderson et al., 2014). Another study in the same species further revealed the importance of juvenile experience. In that study, females were collected from the wild as adults and tested in the laboratory for their preference for songs recorded from either their local breeding population or from another population over 500 km away (Anderson, 2009). Initial tests revealed that females expressed a strong preference for songs from their local population. The same females were then provided with extensive exposure to songs from the distant population, and then the females were tested again. Even after extensive and exclusive exposure to songs from the more distant population, the females’ preference for songs from their local breeding population remained stable (Anderson, 2009). Together, these results indicate that female preference in this species is strongly influenced by developmental auditory experience and is much less vulnerable to the influence of adult auditory experience.

In other species, females can modify their song preferences to include songs that they heard only later in life. For example, reports have documented that females of many species prefer songs that they heard during juvenile development over songs performed by males that they encountered only in adulthood and are therefore novel to them at the time of testing (King et al., 1980; Baker et al., 1987; Clayton, 1990; Searcy, 1990; Searcy et al., 1997, Searcy et al., 2002; Freeberg et al., 2001). However, when adult zebra finch females are provided with extensive exposure to songs that they had previously never heard, they can expand their preferences to include those novel songs (Clayton, 1988). This pattern is also evident in female canaries and cowbirds, as females typically show a preference for songs that they heard during juvenile development, but their preferences can expand to include songs of unfamiliar males with which they have formed pair bonds and raised successful broods of offspring (Nagle and Kreutzer, 1997; West et al., 2006). When we consider results obtained from many species, a common theme is that females prefer songs from males in their local population and those they experienced during juvenile development. The ability of adult females to modify their preferences seems to vary within a range.

Acquisition of song preferences during juvenile development and possible plasticity of song preference throughout adult life afford many possible advantages and disadvantages. For example, a static preference could facilitate recognition of not only members of the female’s own species, but also members of her home group. This could lend stability to populations even if home ranges become disrupted or otherwise displaced. Such a pattern could also confer disadvantage in that it could restrict dispersion into new areas and integration with other populations. This could place constraints on genetic diversity and the ability of a group to engage in adaptive behaviors such as hybridization. In contrast to preferences that are static throughout life, dynamic preferences could enable adaptation to emergent conditions such as dispersal by unusual influences, such as storms or errors in migratory navigation. Preferences that can be enlarged through adult auditory and social experience could provide a greater potential for reproductive success and thereby provide a potential mechanism to facilitate genetic diversity and population success.

Many researchers have been intrigued by the ability of different species of songbird to modify their behavior to various degrees in response to adult experience. This is evident in female birds, and male birds may also continue to modify their songs throughout adulthood. For example, males of some species are very sensitive to the influence of auditory experience during juvenile development but are much less vulnerable to the influence of songs that they encounter during adulthood (reviewed in Mooney et al., 2008). These species are said to be “closed learners” and are commonly studied to identify neural mechanisms that enable birds to be so pliable during development and yet so persistent in their skill throughout adulthood. Males of other species are sensitive to the influence of auditory experience during both juvenile development and adulthood. For example, mockingbirds are able to imitate sounds that they hear at any point throughout their lives. These species are called “open ended learners” and offer opportunities to study the neural mechanisms of learning as it occurs in both the juvenile and the adult physiological conditions (reviewed in Mooney et al., 2008). Taking a broad perspective and incorporating results from both types of learners provides an opportunity to investigate the neural mechanisms at work in both conditions (reviewed in Murphy et al., 2017; Schmidt, 2010). Such experiments can address many fascinating questions such as whether adult learning recapitulates the phenomena of juvenile learning, or whether adult learning relies on a different set of mechanisms so that skills and experiences acquired in juvenile learning are preserved while also permitting other forms of behavioral plasticity.

As researchers have looked more closely and more expansively across species, a realization has emerged that learning is more evident across species than was previously appreciated. Even in species in which song was thought to be an innate behavior and the male did not rely on auditory experience to learn the sounds that compose their songs, closer inspection revealed that songs performed by males of those species contain small elements that vary as a function of experience (Saranathan et al., 2007; Kroodsma et al., 2013). Thus, the ability to learn throughout ontogenetic experience appears to be more akin to differences along a spectrum rather than any categorical distinctions between learning versus non-learning species. Incorporating a comparative approach into future studies of how males acquire their songs and how females acquire their song preferences will afford many opportunities to study the possible contributions of a wide range of factors such as experience, genetics, endocrine influences, and especially the neural mechanisms that underlie that behavior (Murphy et al., 2017, Murphy et al., 2020).

Learning in males is closely associated with a network of neural structures called the “song system.” The acquisition of female preferences during juvenile development is reminiscent of song learning that occurs in males, but structures that compose the song system are typically quite atrophied or entirely absent in female birds (reviewed in Mooney et al., 2008). For many years, this led to the impression that song was relatively uncommon among female birds, but more recent studies have indicated that females having the ability to sing either solo or as part of a duet is much more common that was previously appreciated (e.g., Odom et al., 2014; Elie et al., 2019). These new insights into the neurobiology of song perception in female birds have heightened interest in the question of how females acquire their preferences, store those perceptions and preferences in long-term memory, and then recall those experiences in adulthood and use those memories to modify ongoing behaviors. The link between learning and preferences helps elevate the importance of preference research to understand learning. Many researchers focus on male songbirds’ song-learning process to help understand the neurological building blocks of learning. Because female songbirds’ song preferences and mate choice are also influenced by acoustic and social experience, they offer a unique model to study how learning can affect the process of decision making. Researchers have focused on that question much more in recent years. As elaborated in the following section, results have begun to reveal brain sites and pathways that underlie female learning, memory, and behavioral expression of preference for specific songs and the associated singers.

#### 3.2.2 Neural Mechanisms

Behavioral experiments have made it clear that experience plays an important role in shaping those preferences. Here, we turn to consideration of the neural mechanisms that are also shaped by that experience and that underlie the perception and expression of mate preferences. Electrophysiological recordings and pathway tracing studies from many research groups have revealed pathways through which auditory experience is relayed from the ears to the brain (reviewed in Dunning et al., 2018; Bloomston et al., 2022). Activity originating in hair cells of the inner ear is propagated through a network of auditory neurons in the brainstem and thalamus, and it eventually reaches a forebrain area which is the avian analog of the mammalian primary auditory cortex (Field L) (Lewicki and Arthur, 1996; Grace et al., 2003; Amin et al., 2004; Meliza and Margoliash, 2012; Prather, 2013). This first post-thalamic processing region is thought to contribute to the neural basis of natural sound recognition. Electrophysiological recordings in female zebra finches have revealed that Field L neurons respond more robustly to unfamiliar conspecific songs as compared to white noise or synthetic sound stimuli (Hauber et al., 2007). These findings are consistent results from studies of male zebra finches and other songbird species (Leppelsack and Vogt, 1976; Grace et al., 2003). Together these results indicate that at this stage of the auditory processing pathway there may be similar mechanisms that are present in both sexes.

Field L is typically split into three subsections: L1, L2 and L3. L2 is the thalamorecipient portion of Field L, and L1 and L3 relay information to higher auditory areas such as the caudal mesopallium (CM; specifically the medial portion of CM called CMM) and the caudal nidopallium (NC; specifically the medial portion of NC called NCM) (Gobes et al., 2010). Because of these patterns of connectivity, portions L1 and L3 are sometimes considered hierarchically more advanced in the sound processing pathway. Ascending through that pathway, auditory responses become progressively more selective to specific elements of songs and calls (Terleph et al., 2007; Meliza and Margoliash, 2012). CM and NC are secondary auditory processing regions analogous to layers II and III of the mammalian auditory cortex (Karten, 1991). These two forebrain regions are sometimes collectively called the auditory lobule (Cheng and Clayton, 2004; London and Clayton, 2008) and their medial portions (CMM and NCM) have been implicated as potential contributors to formation and storage of auditory memories in male songbirds. Studies of female birds have extended that idea by also implicating them in the expression of learned song preference in females (Riebel, 2003). These and other studies have implicated several brain sites in affecting female song preference, giving rise to additional questions about the neural circuits through which females perceive and evaluate the quality of male song.

Investigations into female song evaluation originated as lesion studies performed using female canaries (*Serinus canaria domestica*). Lesions placed in an auditory-vocal forebrain region known as HVC resulted in a change in female song preference (Brenowitz, 1991). The lesions in that study were quite robust and covered not only most of HVC but also parts of other adjacent auditory-responsive areas including CM and NC. At the time of those experiments, those areas were not appreciated as potentially important in female song perception, and those authors did not control for the possibility that impact of their lesions on sites other than HVC may have also contributed to the observed change in female song perception. A subsequent study of song preference in female zebra finches addressed that limitation by placing smaller and more focal lesions in either HVC or CM. Those authors found that lesions in HVC did not alter song preference. Instead, it was damage to CM that resulted in altered song preferences (MacDougall-Shackleton et al., 1998). Despite those authors’ increased focus on placing small lesions, some of their lesions were so large that they extended beyond the border of CM, thus also impacting regions outside of CM. Moreover, those lesions were generated electrolytically, thus damaging not only somas located within CM but also fibers of passage that course through CM and may need to be intact in order to establish females’ mate preferences. Although these studies have their caveats, they collectively pointed to CM as likely playing an important role in processing auditory signals=.

Additional investigations of CM have revealed pathways through which neurons in that site may communicate with downstream areas to exert their influence on song preference and mate choice. Dunning and colleagues (Dunning et al., 2018) used anterograde tracers to identify the pathways through which CM projects to downstream targets in female Bengalese finches. They found broad agreement between pathways present in females and those previously reported for males (Vates et al., 1996; Mandelblat-Cerf et al., 2014). CM projects robustly to other portions of the auditory lobule (NC). CM also receives small amounts of reciprocal input from NC, as evident in some reports that reveal weak projections from NC to CM and others that detected no connection between those areas (Vates et al., 1996; Dunning et al., 2018; Bloomston et al., 2022). Dunning et al. (2018) also found that CM projects into pathways that provide dopaminergic input back to cortical areas (ventral portion of the intermediate arcopallium, AIV). AIV in male songbirds has been shown to play a role in vocal-motor learning, and is likely a key region for establishing motivational state, which is important for reinforcement learning (Mandelblat-Cerf et al., 2014). In males, AIV provides a connection to midbrain dopaminergic regions, namely the ventral tegmental area (VTA) and the substantia nigra pars compacta (SNpc) (Mandelblat-Cerf et al., 2014). This projection may also play a key role in learning of song and mate preferences in females. Together, these pathways provide mechanisms through which information in CM could be integrated with activity in other auditory processing sites as well as pathways implicated in behavioral motivation and reward.

Dunning et al. (2018) also described two novel pathways emerging from CM in female birds. The first of these is a projection from CM to a site implicated in production of behavioral responses (robust nucleus of the arcopallium, RA). A projection from CM to RA provides a link between a site implicated in auditory perception and downstream sites implicated in production of behavioral indicators of a female’s degree of song preference. Specifically, RA is implicated in the production of calls, which are a way that females indicate their preference for specific songs (Dunning et al., 2014). RA also projects to the midbrain dorsomedial nucleus of the intercollicular complex (DM) (Wild, 1993; Tobari et al., 2006), where activity is both necessary and sufficient for the production of calls (Simpson and Vicario, 1990; Fukushima and Aoki, 2000, Fukushima and Aoki, 2002). DM also projects to the shell of the auditory thalamic nucleus (ovoidalis; Ov_shell_), which then projects to the ventromedial nucleus of the hypothalamus and the mediobasal hypothalamus (MBH) (Durand et al., 1992; Cheng and Peng, 1997). Investigations using measures of immediate early gene expression in female canaries reveal a relationship between levels of activity in CM and MBH in response to songs females find attractive (Cheng and Zuo, 1994; Monbureau et al., 2015). Finally, DM also projects to a medullary respiratory center (nucleus retroambigualis, Ram) which then projects to motor neurons in the spinal cord that control muscles in the cloaca through which females engage in copulation (Wild and Botelho, 2015). This pathway from CM to RA and beyond provides a route through which auditory stimuli may drive expression of behavioral indicators of song preference and mate choice.

The second novel pathway described by Dunning et al. (2018) was a connection between CM and the caudal striatum (CSt). Reiner and colleagues reported that CSt shares characteristics with an auditory region of the mammalian striatum which receives auditory input from Layers 2 and 3 of the auditory cortex (Reiner et al., 2004). This finding points to a role for the auditory areas CM and NC in higher order processing of complex auditory stimuli. The striatum has also been implicated in behavioral selection in mammals (Jiang and Kim, 2018; Cox and Witten, 2019), suggesting another possible route through which activity in auditory cortical areas may influence expression of selective behavioral responses to one suitor among many. This possibility is supported by data showing that optogenetic stimulation of CM can induce dramatic changes in female expression of song preference (Elie et al., 2019). Thus, CM and its projections play a key role in regulating the expression of those behaviors. Future studies will seek to discern the respective contributions of each of the many pathways that emerge from CM.

The reciprocal connections between CM and NC suggest that both sites may contribute to female song preference and mate choice. In support of a role for NC in auditory perception, previous studies of NC have revealed that following lesions to that area there is a reduction in preference for familiar (tutor) songs (Gobes and Bolhuis, 2007). Consistent with this possible role for NC activity in coding familiarity, activity of individual NC neurons habituates rapidly in response to repeated song playback, especially when those song stimuli are conspecific songs as opposed to heterospecific songs (Chew et al., 1995). Additional studies using female zebra finches have shown that when NCM is temporarily inactivated using the sodium channel blocker lidocaine, females show decreased affiliative behavior with conspecific males (Tomaszycki and Blaine, 2014). Taken together, these results indicate that NC may provide a site through which activity in CM may influence perception of song stimuli and the associated expression of affiliative social behaviors. Further insight into these circuits and the cellular mechanisms that compose them are the focus of ongoing research.

## 4 Juvenile Song Preference

### 4.1 Ultimate Questions (Functions)

#### 4.1.1 Song Production Learning

Songbirds acquire their song through vocal learning. The process of song learning has been well documented in some species such as white-crowned sparrows, zebra finches, Bengalese finches, and canaries (Brainard and Doupe, 2002; Okanoya, 2004b; Prather et al., 2017). Although there is diversity in this process (Brenowitz and Beecher, 2005), song learning is commonly characterized by 2 phases: sensory learning and sensorimotor learning (Brainard and Doupe, 2002). Young birds hear and memorize songs of adult conspecifics (song tutor) in the sensory learning phase, and practice singing to match their own vocalization to the memorized song model in the sensorimotor learning phase. Because song is an important courtship signal, choosing an appropriate conspecific song model should be critical for reproductive success. If a juvenile has the opportunity to hear more than one song during sensory learning, how does the bird choose which song to imitate? One possibility is that song preference in juveniles plays a role to guide song tutor choice.

Some species of birds share habitat with phylogenetically close heterospecifics or live in a large flock of conspecifics. In such cases, young birds may hear multiple songs from conspecific and heterospecific adults, but they usually do not learn them all (Mann and Slater, 1995; Takahasi et al., 2010; Peters and Nowicki, 2017). A series of pioneering studies in chaffinches and white-crowned sparrows demonstrated that juveniles selectively learn songs of their own species (Thorpe, 1958; Marler and Peters, 1977; Soha and Peters, 2015; Soha, 2017). When hand-reared juveniles were tutored with playbacks of both conspecific and heterospecific songs, they typically produced species-specific sound features as adults (Marler, 1970). Based on these findings, it was hypothesized that juvenile birds have an auditory template that guides them to pay attention to conspecific song in the sensory learning (Marler, 1970, Marler, 1999). At that time, however, the auditory template was assumed to be some set of mechanisms in the juvenile’s brain but not directly observed either as neuronal or behavioral responses to songs. More recent studies have revealed neuronal responses to juvenile experience that persist even in the adult brain, but they leave open the question of how the template may exert its influence on learning (Prather et al., 2010; Moseley et al., 2017).

In the classical studies, where a disposition in song learning was measured by the consequent song output, there was a methodological problem that one cannot tell if the selective learning is a consequence of perceptual preference of the learner (Rodríguez-Saltos, 2017). Indeed, species-specific physical properties define the range of sounds that juveniles learn to produce, and there should also be interaction between such motor constraints and perceptual preference. However, there is empirical evidence suggesting that juveniles do have preferences in a sense of selective behavioral responses to song stimuli, and such preferences are mostly consistent with the previously found selectivity of song learning. For example, juvenile white-crowned sparrows emit more calls in response to playbacks of conspecific song than to heterospecific song (Nelson and Marler, 1993). Moreover, they acquire memories of specific tutor songs and vocalize more to the familiar songs over unfamiliar conspecific songs (Nelson et al., 1997). Similarly, song-naive zebra finches juveniles prefer their own species song compared to starling songs, when measured by operant behavior associated with song playbacks (Braaten and Reynolds, 1999). In zebra finches, preference for a tutor song (typically father’s song) is also demonstrated with phonotaxis and vocal response assay (Clayton, 1988) as well as key pecking operant conditioning (Rodríguez-saltos et al., 2021). Selective approaches and vocal responses to father’s song was also reported in juvenile Bengalese finches (Fujii et al., 2021).

These findings collectively indicate that male juveniles can discriminate and probably be attracted to the song that they are more likely to imitate. However, in the studies above, the scope was limited to the song preference itself, so the relationship between preference and song learning was not examined. Strict demonstration of the causal relationship between song preference and song imitation learning may be challenging, but we can at least make the following prediction. If juveniles selectively learn the song that they prefer, there should be a positive correlation between the behaviorally measured song preference and the performance of song imitation learning. By testing this prediction, researchers can evaluate the validity of this hypothetical function of song preference. As such, in relatively early years, two studies in zebra finches recorded both the preference and song learning performance (Houx and Ten Cate, 1999; Terpstra et al., 2004). In these studies, preference was tested for the tutor song compared to unfamiliar conspecific song using operant conditioning, but the preference tests were conducted in adulthood, where the birds already finished song learning. Both studies found that the males preferred their tutor’s song over unfamiliar song on average. However, when they examined the relationship of the degree of preference and the tutor-tutee song similarity, one study reported no significant correlation (Terpstra et al., 2004), and the other had a mixed results depending on the condition of song tutoring (Houx and Ten Cate, 1999).

In more recent years, some researchers investigated how social interaction with the song tutor enhance song learning in juveniles (Chen et al., 2016; Baran, 2017). In these studies, the authors did not measure the song preference itself but showed interesting relationships between the behavior of juvenile learners during song tutoring and the result of song production leaning. For example, juveniles generally pay closer attention to song when performed by a live tutor than when played through a speaker, and the degree of attention towards the song during tutoring predicts the accuracy of song imitation (Chen et al., 2016). In this study, attentiveness was defined by the lack of engagement in other behaviors such as feeding, flying, or vocalizing. Another study pointed out a correlation between social attachment to parents and song copying from the father in juvenile zebra finches (Baran et al., 2016, Baran et al., 2017). Although the relationship among the preference, attention, and social interaction is still unclear, this line of research may shed light on the possible function of song preference in sensory learning.

We also note here that preference for tutor song may help not only sensory learning but also sensorimotor learning. In a recent study using zebra finches, the authors quantified juveniles’ approach to their singing tutor and found that individual variability of approach behavior correlated with the precision of tutor song imitation (Liu et al., 2021). They proposed a two-step auditory learning process; juveniles first acquire a crude memory of their tutor song, which drives repeated interaction with the singing tutor, leading to ensure the selective and precise vocal imitation. As they did not compare the juveniles’ response among different songs or song tutors, the degree of attentive listening behavior may not be directly equivalent to song preference as measured in other studies. However, it suggests a possibility that early acquisition of preference for the song of an individual tutor may function to further reinforce accurate imitation.

Regardless of which phase of learning the conspecific or tutor song preference may serve, crucial empirical evidence for the causality between preference and song production learning is still lacking, either at behavioral or neural levels. Yet, a breakthrough may be provided by cutting-edge studies. By using a combination of carefully controlled live tutoring and well-designed operant conditioning preference tests, it was recently reported that song preference of juveniles in song development predicts the quality of future song imitation (Rodríguez-saltos et al., 2021). Another study from the same group using the same experimental system attempts to reveal the neural mechanisms underlying the tutor song preference and vocal learning (Pilgeram et al., 2021).

#### 4.1.2 Other Possibilities

There are also other possible functions of song preference in young birds. One example is parental recognition. Because of the entire nutritional dependence on parental care in young altricial songbirds, correct recognition of the species or identity of adult birds should be critical to the survival of birds at a very early developmental stage. Some studies have shown that nestlings (Shizuka, 2014; Wheatcroft and Qvarnström, 2017; Hudson et al., 2020) and young fledglings (Nelson and Marler, 1993; Whaling et al., 1997; Soha and Marler, 2001) respond to conspecific songs with more calls than to heterospecific songs. These results support the idea that the preference may help very young birds correctly recognize their parents and beg for food or care. However, it should be noted that parental recognition can also be accomplished through other sensory domains. For example, nestling zebra finches can use odor to detect the presence of their parents and initiate begging behavior (Caspers et al., 2017). In addition, it is likely that young birds use vocalizations other than song in species where only males sing, as biparental care is a typical characteristic of songbirds in either group of female song present or absent (Langmore, 1998; Cockburn, 2006). Thus, future studies are awaited to figure out whether young birds use their parents’ (or only father’s) song as a cue for parental recognition.

Finally, we add some notes for fair estimation of the utility of juvenile song preference. First, the fact that young birds respond differently to song stimuli in experimental settings does not necessarily indicate that the birds do utilize such discrimination ability in natural social settings (Nelson and Marler, 1990). In addition, it is also possible that birds acquire song preference when young, but the preference comes to have its utility only after the birds become adult. In such a case, even if the song preference is not in effect, it can be observed in young birds if researchers attempt to do so. Conversely, juvenile song preference can also have multifaceted functions, thus different functions described above are not mutually exclusive. Lastly, as we currently know very little about the phylogeny of juvenile song preference, more comparative studies are expected in the future.

### 4.2 Proximate Questions—Mechanisms and Development

Although we currently know less about the neural mechanisms underlying song preference of juveniles compared to that of females, there are a substantial number of neurobiological studies suggesting the relevance of higher auditory forebrain areas including NCM. This auditory region originally attracted attention because evidence indicated that it is the locus of tutor song memory as a template of imitative song learning (Adret, 2004; Bolhuis and Gahr, 2006; Hahnloser and Kotowicz, 2010; Bolhuis and Moorman, 2015, but see also; Ikeda et al., 2020). It is still one of the hottest topics in the songbird neuroscience, and the role of forebrain auditory areas in song production learning is well discussed in the reviews listed above. Here, we focus on the literature that is particularly related to the preference for conspecific songs or the tutor song.

It was first found in adult songbirds that NCM exhibits stimulus-specific neural responses (Mello et al., 1992; Chew et al., 1996; Bolhuis et al., 2000; Phan et al., 2006). Later, mainly in zebra finches, the neural responses were compared across ages to investigate how such neural selectivity develops. Consistent with the sensitivity to own-species song at a behavioral level, electrophysiological recordings (Stripling et al., 2001; Schroeder and Remage-Healey, 2021) and immunohistochemical assay (Stripling et al., 2001; Bailey and Wade, 2003, Bailey and Wade, 2005) showed that some neurons in the NCM selectively respond to conspecific song over heterospecific song either at a single-cell level or at a population level. These results indicated that the selectivity is already present at the age of 20 days post hatch, which is said to be the beginning of the sensory learning in this species (Roper and Zann, 2006). Similarly, it was also found that a population of NCM neurons selectively respond to tutor song among other unfamiliar conspecific songs. The hypothesis that the NCM neurons code the tutor song memory was originally suggested by a series of findings in adult birds (Bolhuis et al., 2000, 2001; Terpstra et al., 2004; Phan et al., 2006), but recent electrophysiological studies showed that tutor song selective neurons are present at an early stage of sensory learning but may be small in number (Miller-Sims and Bottjer, 2014), and that such selectivity is actually shaped through auditory experience of tutor song (Yanagihara and Yazaki-Sugiyama, 2016; Moore and Woolley, 2019). It is not clear, however, whether these neural activities are the basis of tutor song preference. To our knowledge, there has been no study that impaired the neural activity of NCM in juveniles and examined the effect of impairment on the song preference, although one study reported that NCM lesion in adult male zebra finches impaired the tutor-song selective approaching behavior (Gobes and Bolhuis, 2007).

Another line of studies suggests the importance of midbrain catecholaminergic systems during song tutoring. For example, in male juvenile zebra finches, noradrenergic neurons in the locus coeruleus as well as dopaminergic neurons in VTA show activities correlated with attention to tutor song. Behaviorally measured attentional state and the neuronal responses in these midbrain areas both increased during live tutoring compared to playback tutoring (Chen et al., 2016). Also, a recent study from the same group showed that noradrenaline release in NCM during social tutoring is important for the formation of tutor song memory (Chen and Sakata, 2021). Another study reported that pharmacological manipulation of the dopaminergic system in juvenile zebra finches changed the approaching behavior towards tutor songs, and that activation of immediate early gene in the NCM in response to song playbacks was associated with the listening approaching behavior of the juveniles (Liu et al., 2021). Thus, the noradrenergic and dopaminergic systems seem to be involved in the mechanism of juvenile song preference, though the specific contributions of these systems in the expression or plasticity of preference are currently unknown.

## 5 Discussion

The results of studies focusing on birdsong preference have accumulated and now researchers have a basis to develop a more precise investigation into its neural mechanisms. In this section, we describe some problems that should be kept in mind when further exploring both the proximate and ultimate explanations for song preference and discuss possible future directions.

### 5.1 Choice and Validation of Behavioral Index of Preference

When choosing a method, it is important to balance the practicality and ecological validity. Indeed, if a bird consistently shows different degrees of a given behavioral response to different songs, it means that the birds can discriminate between stimuli. However, whether the discrimination is interpreted as the preference in the context of mate choice or song tutor choice is not clear until additional tests can verify the ecological validity of the behavior observed in the tests. We believe this is a critical issue for any behavioral and neurobiological laboratory study. Without the firm understanding of the focal behavior, one can lose sight of the mapping between neural activity and natural behaviors (Krakauer et al., 2017).

Here we take call back assay as an example. Although the method has been used to test preferences in females and juveniles of some species (Clayton, 1988; Nelson et al., 1997; Chen et al., 2017), it was not a standard procedure to differentiate the categories of calls with consideration of the function of each call type (but see Nagle et al., 2002). Of course, such a fact does not necessarily deny the reliability of the assay. Nonetheless, birds usually communicate with others in various behavioral contexts through different categories of calls (Marler, 2004; Elie and Theunissen, 2016), and calls are sometimes regarded as an indicator of the more general attentional state such as familiarization to stimuli (Dong and Clayton, 2009; Ono et al., 2015; Dai et al., 2018). Probably, the best way to verify the calls as a measure of preference would be to carefully observe the animals’ behavior in natural social settings. As this would not always be very practical, an alternative way is to examine the correlation between behavioral indices in laboratory experiments. As such, in a series of studies of female Bengalese finches, the response rate of a specific call type was positively correlated with CSDs as well as with phonotaxis at an individual or a population level (Kato et al., 2010; Dunning et al., 2014; Fujii et al., 2021; Fujii and Okanoya, 2022). Because using multiple behavioral tests to measure subjects within a single study can be logistically challenging, the most practical approach may be to gradually accumulate the results from an entire research community over time. This would also enable researchers to examine the reproducibility of results obtained from the same experimental system, as well as the consistency of results between different types of experiments. This cycle of careful reconsideration and application of methods should also be vital to evaluate the impact of neural manipulation on song preferences.

The choice of behavioral index may be critical in figuring out the causal relationship of juvenile song preference and vocal learning. When investigating the correlation between behaviorally expressed preference and the results of song learning, do differences in the choice of behaviors lead to different results? To date, some studies utilized operant conditioning to quantify the tutor song preference (Terpstra et al., 2004; Pilgeram et al., 2021; Rodríguez-saltos et al., 2021), while others measured birds’ activities including approaching behavior as an indicator of stimulus engagement or attentional state (Chen et al., 2016; Liu et al., 2021). Currently, we do not know whether these behaviors capture the same aspect of song preference or whether they share the neural basis. In future studies, identifying the neural substrate responsible for juvenile song preference and testing the effect of manipulation of the preference-related neural activity on the performance of vocal imitation would be one of the most powerful approaches to examine the function of preference in learning.

### 5.2 Inter- and Intra-Species Variation of Song Preference

Another important perspective is the variation of song preferences. As seen in the diversity of song characteristics across species, there is great species-diversity in the function, evolutionary background, and the developmental process of songs (Brenowitz and Beecher, 2005; Catchpole and Slater, 2008). This diversity means that we cannot necessarily generalize the knowledge of one species to another, and thus a comparative viewpoint is needed for a comprehensive understanding of any aspects of birdsong, including how and why females or juveniles prefer a certain song over others. In a case where many different species of birds possess a similar profile of preference, either by convergent evolution or inheritance from a common ancestor, studying one representative model species helps construct a general understanding. However, the postulation itself is the very subject of scientific examination, and there has been a bias in the choice of subject species particularly in the neurobiological laboratory experiments; zebra finches are the most studied species, and other birds such as Bengalese finches and cowbirds follow. Even the few species listed here show great diversity in their habitat, life history, or evolutionary background (Okanoya, 2004b; Perkes et al., 2019; Griffith et al., 2021), further advancing the existing research topics in these species and others is quite worthwhile. However, direct comparison of a limited number of species of great phylogenetic distance is rather difficult and may not be relevant. Thus, extending the scope to more diverse taxa of songbirds as well as comparing multiple species of close phylogenetic relationship would be meaningful in future studies.

Individual difference of preference within a species should also be considered as a direction to extend research efforts. The importance of individual variation has already been pointed out particularly for the study of mating preference; investigation into the basis of variation in mating preferences has the potential to reveal new insights into the evolutionary history of sexually selected signals and the signal preference itself (Jennions and Petrie, 1997). We believe this is also a context in which Tinbergen’s four questions can interact in a fruitful way. For instance, in the studies on Bengalese finches from our laboratories, we found that while some song features are preferred by majority of females, there are also substantial individual differences in the song preference (Morisaka et al., 2008; Kato et al., 2010; Dunning et al., 2014, 2020). Other studies provide a possibility that the source of individual variation lies in the early-life experience of father’s song (Fujii et al., 2021; Fujii and Okanoya, 2022). As the Bengalese finch song is particularly interesting for the sequential complexity and its evolutionary change through domestication process (Okanoya, 2004a; Okanoya, 2004b), scrutinizing the universality and individual differences of female song preference from the perspectives of neural mechanisms and development potentially help investigate the function and evolution of the sequential complexity, and vice versa. This is also the case for song preference of juveniles, as they have a potential to filter the song features transmitted to the next generation by selective imitation (Soma et al., 2009; Peters and Nowicki, 2017).

### 5.3 Neural Basis of Song Preference

The neuroscience of female song preference began with the exploration of brain areas involved in the expression of preference using electrolytic or pharmacological lesions as well as correlation analysis between the neural and behavioral response selectivity to song stimuli. The results from recent neural tracing and imaging studies (Dunning et al., 2018; Van Ruijssevelt et al., 2018; Perkes et al., 2019), together with the techniques of precise manipulation of neural activities (Elie et al., 2019; Barr et al., 2021) now provide a prospect of investigation into the mechanisms of song preference at a circuitry level. To elucidate the functional roles of each component in the proposed neural circuits for the expression of song preferences (Dunning et al., 2018; Van Ruijssevelt et al., 2018; Perkes et al., 2019), optogenetic manipulation of specific neuronal populations with a fine anatomical and temporal resolution would be a powerful tool (Elie et al., 2019).

The neural mechanisms underlying plasticity of song preference is also worth studying. Strong influence of postnatal experience on the signal preference is one of the most interesting features in songbirds compared to other vertebrate or invertebrate taxa (Riebel, 2009; Verzijden et al., 2012), and experience-dependent changes in song preferences can occur throughout life (Riebel, 2009). Recent studies in adult female zebra finches showed the involvement of the dopaminergic system in the acquisition of song preference using pharmacological manipulation (Day et al., 2019; Barr et al., 2021), but the neural mechanisms by which song preference is shaped early in development is still unclear. It would be interesting to examine the degree to which the neural circuitry composed of higher auditory forebrains and dopaminergic neuromodulation also plays a role in the brain of juveniles similarly to that of adults.

We also note that the inter-species comparison of auditory neural response properties may shed light on the evolutionary process that have shaped song preferences. Although the evolutionary mechanisms of bird song preference other than fitness benefits remain poorly understood (but see Collins, 1999; Eda-Fujiwara et al., 2006), such a comparative approach succeeded in anurans to find the significant role of sensory bias in the preference of acoustic signals (Ryan and Cummings, 2013).

In a case of male juvenile songbirds, the neuronal activities in the forebrain auditory area NCM have been explored in search of the neural substrate of tutor song memory used for vocal imitation (Bolhuis and Gahr, 2006; Bolhuis and Moorman, 2015). It is known from classical studies that male juveniles have a disposition to selectively learn conspecific songs. More recent behavioral and neurobiological studies suggest the relevance of juveniles’ perceptual preference among conspecific songs (Fujii et al., 2021; Liu et al., 2021; Pilgeram et al., 2021; Rodríguez-saltos et al., 2021) as well as the importance of juveniles’ physiological or arousal state during the exposure to the song they are about to learn (Chen et al., 2016; Yanagihara and Yazaki-Sugiyama, 2019; Chen and Sakata, 2021; Liu et al., 2021). These researchers focus on NCM, while also indicating the involvement of the midbrain catecholaminergic system in the processes of memory formation or increased attention to the tutor song. To date, we do not know if there are distinct neural substrates of song preference and song template within NCM. Attempts to experimentally dissociate the two kinds of neural representation will be challenging, but this direction of neurobiological research should also be helpful to reveal the relationship of preference and song learning at a behavioral level. In addition, it is not known if the midbrain catecholamine neurons differentially respond to more preferred or less preferred songs. Moreover, it needs to be examined if the catecholaminergic signaling is relevant not only to the formation or plastic changes of song preference but also to the expression of preference. Future studies might ask to what extent the neural activity correlated with behavioral song preference depends on previous auditory experience. It would be important to figure out whether certain neural response properties are actually the basis of behavioral preference for a song with specific features, and whether they guide song tutor choice.

### 5.4 Summary and Conclusion

Song preference of female and juvenile songbirds has been studied as an important factor in mate choice and song tutor choice, respectively. Based on the previous ethological findings, researchers are now investigating the neural mechanisms underlying the representation, expression, and plasticity of song preferences. The higher auditory forebrain areas and the midbrain catecholaminergic systems are of particular interest in both cases of females and juveniles. For quantification of the preference in the neurobiological laboratory research, experimenters need to carefully choose a method of behavioral test, balancing the practical use and ecological validity. We suggest that incorporating the knowledge about ultimate questions of songs and song preferences is helpful in doing so. Conversely, the future exploration into the proximate questions of song preference would shed light on the function and evolution of the preference, leading to more comprehensive and general understanding of a complex nature of vocal behavior. The multifaceted approach to the questions of birdsong preference will potentially give insight into the study of speech communication in humans.
